# Physicochemical and Textural Features of the Shuidong Mustard (*Brassica juncea*) with a 15-Day Microorganism Fermentation Under a Lower Table Salt Usage

**DOI:** 10.3390/foods15071185

**Published:** 2026-04-01

**Authors:** Ming-Yue Zhong, Ya-Zhu Xiao, Qing-Qi Guo, Xin-Huai Zhao

**Affiliations:** 1 School of Biology and Food Engineering, Guangdong University of Petrochemical Technology, Maoming 525000, China; 13644385082@163.com; 2 College of Life Sciences, Northeast Forestry University, Harbin 150040, China; 16645121469@163.com; 3 College of Food Science, Northeast Agricultural University, Harbin 150030, China

**Keywords:** Shuidong mustard, fermentation, chemical composition, microorganism structure, texture, volatile compound

## Abstract

In the present study, Shuidong mustard (*Brassica juncea*) produced in Maoming City, Guangdong Province, was fermented at 25 °C for 15 days, using wild microorganisms and 20 g/kg table salt in water. The results showed that this fermentation endowed Shuidong mustard with acid production via utilizing reduced sugar as the fermentation substrate, causing the fermented Shuidong mustard to have a decreased pH value and increased total titratable acidity. Partly as the result of NaCl usage or fermentation, the fermented Shuidong mustard had enhanced NaCl or ash contents, decreased contents in nitrite/nitrate, vitamin C, total phenols, and total carotenoids, and altered textural features reflected as reduced hardness, chewiness, springiness, and fracturability. Moreover, 90 volatile compounds, including 2-butyl, 3-butenyl, isobutyl, and ethyl isothiocyanates, were detected in the fermented Shuidong mustard after the 15-day fermentation, while 21 members comprised 95% (*w*/*w*) of total volatiles. Additionally, the analysis results revealed that the microorganism community of fermented Shuidong mustard was structured at respective phylum, genera, or species levels by *Firmicutes* and *Proteobacteria*, or *Lactiplantibacillus*, *Enterobacteriaceae*, *Lactococcus*, and *Pediococcus*, or *Lactiplantibacillus*, *Enterobacteriaceae*, *Pediococcus*, and *Lactococcus*. It is thus concluded that this explored fermentation induced both acid production and, more importantly, compositional and textural changes in Shuidong mustard, which had production potential at an industrial scale as part of a healthy diet because these bioactive compounds include isothiocyanates, polyphenols, and carotenoids. Overall, this study focused on the Shuidong mustard fermentation using 20 g/kg table salt to fill a research gap in low-salt fermentation, showing its significance by providing a scientific basis for product development.

## 1. Introduction

As a prevalent method in food processing, microorganism fermentation of foods employs specific conditions to cultivate one or more edible and beneficial microorganisms in food matrices including fruits, vegetables, flours, milk, meat, or their derived products. As a result of microorganism fermentation, the food products obtained have enhanced shelf life and acquire unique sensory characteristics and quality attributes [1]. Globally, the production of fermented vegetables exhibits substantial diversity, shaped by regional traditions and, more importantly, methodological differences employed in vegetable fermentation. Notable international examples of fermented plant foods encompass Korea kimchi, Indian pickles, sauerkraut of German origin, and Russian-style pickled cucumbers and mushrooms [2,3,4]. Moreover, Chinese culinary traditional diets also feature a variety of fermented vegetable items, among which are Sichuan pickles and northeastern sauerkraut. In southwestern China, traditional pickled mustard greens are also produced, while the Jiangxi region is known for its distinctive preserved vegetables [5]. Generally, several vegetables including radish, Chinese cabbage, stem mustard, and mustard greens are served as raw materials in the processing of these fermented products [6,7]. It is now recognized that food fermentation can preserve the key nutritional components of vegetables, while microbial activity can induce the generation of some bioactive compounds with potential health benefits in the body. Fermented vegetable products are thus considered as part of a health-promoting diet. For example, the results demonstrated that the fermented mustard, Chinese cabbage, and slaw possessed an anticancer effect on colon cancers because of the bioactive compounds (e.g., phenolics) produced during the fermentation process [8]. Specifically, these bioactive compounds were regarded as able to improve colonic well-being through regulating intestinal conditions and inhibiting the propagation of cancer cells [8]. Further scientific findings also indicated that the fermented varieties of cabbage, soybean, and radish had physiological or healthy benefits in the body, including the attenuation of aging, modulation of blood pressure, protection against cardiovascular disorders, and reduction in cholesterol levels [9,10,11]. Overall, contemporary consensus holds that food fermentation driven by microbial activity can enhance both the sensory quality and health benefits of vegetable-based fermented products. The researchers thereof are exploring the quality and health functions of the fermented plant foods that are well- or less-known to the public.

To achieve a sufficient preservative effect and flavor development, many fermented vegetables are typically prepared with a high concentration of table salt; that is, NaCl contents in these products are generally maintained at a higher level. Many fermented vegetable products commonly consumed are thus characterized by their high NaCl contents; for example, Korean kimchi, and German sauerkraut [2,3,4]. In China, the fermented mustard tuber, sauerkraut, and potherb mustard typically undergo a dehydration by table salt during the initial fermentation stage (e.g., using NaCl content of 60 g/kg), resulting in these products having elevated NaCl contents [1]. However, such high NaCl intake is considered to contradict with the modern dietary guidelines, which now recommend reduced NaCl intake in daily diet. The level of NaCl intake has been regarded as directly associated with various health risks; for example, excessive NaCl intake is identified as a risk factor for cardiovascular diseases such as hypertension. Excessive Na^+^ can increase osmotic pressure in body fluids, raising blood volume and thereby elevating blood pressure. More importantly, long-term hypertension is observed to accelerate arterial stiffness and increase cardiac load significantly, ultimately raising the risk of cardiovascular events such as stroke and coronary heart disease. Additionally, a high-salt environment can directly damage the gastric mucosal barrier, triggering undesired inflammatory responses and mucosal atrophy [12]. Thus, the regions with frequent consumption of fermented vegetables with high salt levels often exhibit a higher incidence ratio of gastric cancer [13]. To guide the importance of salt reduction in diet, the World Health Organization (WHO) has recommended that daily NaCl intake for adults not exceed 5 g [14]. Based on this consideration, it is necessary to produce fermented vegetables with reduced salt contents through developing new or updated processing techniques.

Traditional methods used in food fermentation are mostly recognized as one of the oldest and most widespread techniques in human history, but recent developments in human nutrition suggest necessary technological improvement; for example, using the so-called “low-salt fermentation” to reduce the usage of table salt. Various salt-reduction strategies thus have been explored in both scientific research and industrial application. In the preparation of northeastern sauerkraut and Sichuan pickles, rapid pH decrease could be achieved through an inoculation with competitive lactic acid bacteria starters, which might inhibit the growth of undesirable microorganisms and then enable dominant fermentation without reliance on high salt concentration [15]. Additional techniques such as phased salt adjustment, vacuum pickling, and ultrasound-assisted processing were also applied to facilitate the fermentation under the reduced salt usage [13]. Interestingly, the application of the low-salt fermentation is also regarded as able to optimize the microbial community and preserve vitamin C content in vegetables [13]. Meanwhile, the core of traditional fermentation depends on the so-called “natural microbiota” or wild microorganisms. Generally, these wild microorganisms, including bacteria, yeasts, and molds, which have been adhered to the surface of raw materials and processing tools or existed in the surrounding environment, are utilized to initiate and complete the targeted fermentation. For example, Jiang and coauthors employed the microbial community in the fermentation of leaf mustard [16]. In the past, researchers also developed various microbial starters and investigated their application potentials in food fermentation [13,14,15,17].

In the present study, one famous vegetable, namely Shuidong mustard (*Brassica juncea*), which is widely planted and consumed in Maoming City (Guangdong Province, China), was explored for its anaerobic fermentation of 15 days with wild (i.e., native) microorganisms under a lower table salt usage (20 g/kg, on the weight basis of fresh vegetable). Several assessments were performed on the fermented mustard samples, while Shuidong mustard with a fermentation time of zero days was used as control, aiming to monitor the acid production and consumption of reducing sugar, clarify the main chemical compositional changes in these major and minor components including moisture, NaCl, ash, vitamin C, total phenols, total carotenoids, nitrite, and nitrate, and identify textural changes using the texture profile analysis (TPA). Meanwhile, the volatile compounds in the fermented Shuidong mustard were briefly identified *via* the headspace solid-phase microextraction combined with the gas chromatography–mass spectrometry (HS-SPME-GC-MS) technique, while the main microorganisms in the fermented Shuidong mustard were also evaluated by the classic high-throughput sequencing technique. The purpose of the present study focused on an investigation about the chemical, textural, and microbiological profiles of the fermented Shuidong mustard when a lower concentration of table salt was used in its fermentation. The present study might reveal whether the explored low-salt fermentation was applicable for Shuidong mustard, and it could provide insights to the fermentation progress of Shuidong mustard and its quality features. Thus, the obtained results might provide scientific basis for the industrial production and market development of the fermented Shuidong mustard with reduced salt content.

## 2. Materials and Methods

### 2.1. Materials and Reagents

Fresh Shuidong mustard was obtained from a market in Maoming City, while the table salt used in fermentation only was purchased from a market in Harbin City (Heilongjiang Province, Chain). All reagents used in the present study, such as diethyl ether, phenolphthalein, 3,5-dinitrosalicylic acid, NaOH, KOH, HCl, phenol, oxalic acid, zinc acetate, potassium chromate, and others, were analytical grade and supplied by Shanghai Aladdin Biochemical Technology Co., Ltd. (Shanghai, China). Distilled water was used in the present study to prepare all solutions.

### 2.2. Fermentation of Shuodong Mustard

The fresh Shuidong mustard was washed with drinking water to remove the dust or other pollutant particulates and then air-dried to remove residual water on the mustard surface. Afterwards, the mustard of 1500 g was equally placed into three fermentation jars (2.5 L volume capacity for each), while each jar was added with 2 L of a saline solution (i.e., cooled sterilized water containing 20 g/kg table salt), which ensured all mustard samples were completely covered by the saline solution. The jars were sealed and kept at an environment of 25 °C for 15 days to perform the targeted mustard spontaneous fermentation. At the fixed fermentation times of 0, 2, 5, 7, 10, 13, and 15 days, the fermented mustard samples were collected randomly and then used for subsequent analyses or evaluations. In detail, the physicochemical and textural indices were determined at the same day, while microbial diversity and volatile compounds were analyzed subsequently [1].

### 2.3. Determination of pH and Total Titratable Acidity

The value of pH for the homogenized mustard sample was determined with a digital pH meter (PHS-3G, Inesa Scientific Instrument Co., Ltd., Shanghai, China). Total titratable acidity was quantified using a titration method and the 0.1 mol/L NaOH solution. Briefly, a homogeneous slurry was prepared by blending 5.0 g of the sample with 5 mL of distilled water, and then it was titrated with the NaOH solution using phenolphthalein as an indicator. Total titratable acidity was estimated as the lactic acid amount in 1 kg of the sample.

### 2.4. Determination of Moisture, Ash, NaCl, Nitrate, and Nitrite Contents

The quantification of moisture and ash contents in the sample was performed using the AOAC Methods 934.01 and 937.09 [18], respectively. Briefly, an appropriate amount of the sample was weighed and dried at 105 °C to constant weight, while the value of moisture content was calculated by mass difference. For the ash determination, the sample was incinerated to constant weight using a 550 °C muffle furnace (SX2-4-10N, Shanghai Yiheng Scientific Instrument Co., Ltd., Shanghai, China). Meanwhile, an ion chromatography (Metrohm 881 Compact IC pro, Herisau, Switzerland) was employed to detect the concentrations of NaCl, nitrate, and nitrite using the established protocols [19,20] and standard curves, respectively.

### 2.5. Determination of Total Reducing Sugar and Vitamin C

Homogenized sample aliquot (5.0 g) was mixed with 10 mL of distilled water and incubated at 80 °C for 30 min, cooled to 20 °C, and centrifuged at 10,000 *g* for 10 min. The supernatant was harvested, while the sample residue was extracted again with 10 mL of distilled water. The combined supernatant was diluted to 25 mL with distilled water, while 2 mL of the diluted supernatant was reacted with 1.5 mL of 3,5-dinitrosalicylic acid solution (10 g/kg) in a boiling water bath for 5 min. The absorbance value at 540 nm was thus recorded *via* a spectrophotometer (UV-2600, Shimadzu Corporation, Kyoto, Japan). Total reducing sugar content was determined based on a fresh prepared standard curve of glucose, as previously described [21].

Vitamin C level within the sample was determined according to a reference method [22]. Briefly, 5.0 g of the homogenized sample was added with 10 mL of oxalic acid solution (2 g/kg), mixed efficiently, and then centrifuged at 10,000 *g* for 10 min. The resulting supernatant was separated and subjected to a titration using a 2,6-dichlorophenol indophenol dye solution (0.05 g/kg), while vitamin C content was calculated as previously described [22].

### 2.6. Determination of Total Phenols and Carotenoids

Total phenol content was determined and calculated as previously described [23], using the Folin–phenol colorimetric method and gallic acid as the standard. Briefly, 5 g of sample was homogenized in 75% ethanol, followed by one-hour ultrasonic extraction. After the centrifugation (12,000 rotation/min, 15 min, 4 °C), 1 mL of the supernatant was mixed efficiently with 6 mL of distilled water and 0.5 mL of 1 mol/L the Folin–Ciocalteu reagent, incubated in darkness for 3 min, added with 1.5 mL of 200 g/kg Na_2_CO_3_ solution, and allowed to stand for a reaction time of 2 h. The absorbance value was recorded at 765 nm by the spectrophotometer to determine total phenol content, which was expressed as mg/kg fresh weight.

For determination of total carotenoid content, the analysis was performed using a method from the literature [23] with β-carotene as the standard. Briefly, 5.0 g of the homogenized sample was mixed with 4 mL of diethyl ether in a 15 mL centrifuge tube, saponified by adding 0.5 mL of saturated KOH solution, kept in the dark for 30 min, added with 5 mL of distilled water, and centrifuged at 10,000 *g* for 3 min. The diethyl ether layer was thus separated and measured for the absorbance value at 450 nm using the spectrophotometer. The value of total carotenoid content was calculated as previously described [23] and then expressed as mg/kg fresh weight.

### 2.7. Determination of Textural Indices by TPA

The textural properties of the sample were assessed with a texture analyzer (CT3 Texture Analyzer, Brookfield Engineering Laboratories, Middleboro, MA, USA) using the stalk section of the prepared sample. A stainless steel probe (a needle-like shape, 1 mm in diameter and 43 mm in length) was employed in this determination. For each sample, ten penetration tests were performed using a test speed of 1 mm/min and a trigger force of 5.0 g, while four textural indices, namely hardness, chewiness, springiness, and fracturability, were estimated from the TPA curve. Following the published study [6], hardness and chewiness were defined as the sample’s resistance to external pressure and the force required for chewing, while springiness and fracturability were defined as the ability of a sample to return to its original shape after compression and its susceptibility to breakage upon impact, respectively.

### 2.8. HS-SPME/GC-MS Analysis of Volatile Compounds

The volatile compounds in the sample were analyzed by the HS-SPME/GC-MS technique, using the sample fermented for 15 days. The analysis was conducted by Hangzhou Kaitai Biochemical Technology Co., Ltd. (Hangzhou, China) using an Agilent 8890A GC system (Agilent Technologies, Palo Alto, CA, USA) equipped with a split/splitless injector and a dual-stage cryogenic modulator (LECO), interfaced with a time-of-flight mass spectrometry (TOFMS) detector. The extraction parameters used in this analysis were identical to those described in a reference study [24]. The identification of these volatile compounds was carried out by matching the obtained mass spectra against the NIST2023 Mass Library Match.

### 2.9. Analysis of the Structure of Microbial Community

The analysis was conducted by Hangzhou Kaitai Biochemical Technology Co., Ltd. (Hangzhou, China), using the sample fermented for 15 days. Sample DNA was isolated using a DNA extraction kit (OMEGA, Norcross, GA, USA) following the supplier’s protocol. Subsequently, full-length bacterial 16S rRNA and ITS rRNA were PCR-amplified using the extracted DNA as template. The V3–V4 hypervariable region of the 16S rRNA gene was amplified with the primers B341F (CCTACGGGNGGCWGCAG) and B785R (GACTACHVGGGTATCTAATCC) in a polymerase chain reaction system from Vazyme Biotech Co., Ltd. (Nanjing, China). Similarly, the ITS1 region of the fungal ITS rRNA gene was amplified by employing primers ITS1-F (CTTGGTCATTTAGAGGAAGTAA) and ITS2-R (GCTGCGTTCTTCATCGATGC). The PCR protocol included the initial denaturation at 95 °C for 3 min, followed by the 37 cycles of denaturation at 95 °C for 30 s, annealing at 55 °C for 30 s, and extension at 72 °C for 10 min, with a final extension at 72 °C for 5 min. The library fragment size was verified by 2% agarose gel electrophoresis, while the concentration was measured using a Qubit 3.0 fluorometer (Thermo Fisher Scientific, Waltham, MA, USA) to ensure uniform clustering and high-quality sequencing. Finally, the sequencing was carried out on the Illumina MiSeq platform (Hangzhou Kaitai Biotech. Co., Ltd., Hangzhou, China).

### 2.10. Statistical Analysis

The data are presented in this study as mean values or mean values ± standard deviations, based on at least three replicate measurements for each sample (with the exception of textural analysis). Statistical comparison among different groups was performed using one-way ANOVA followed by Duncan’s post hoc test in SPSS Version 20.0 (SPSS Inc., IBM, Armonk, NY, USA). A significance level of *p* < 0.05 was applied to determine significant differences statistically.

## 3. Results and Discussion

### 3.1. Acid Production and Consumption of Reducing Sugar During Shuidong Mustard Fermentation

**Figure 1:**
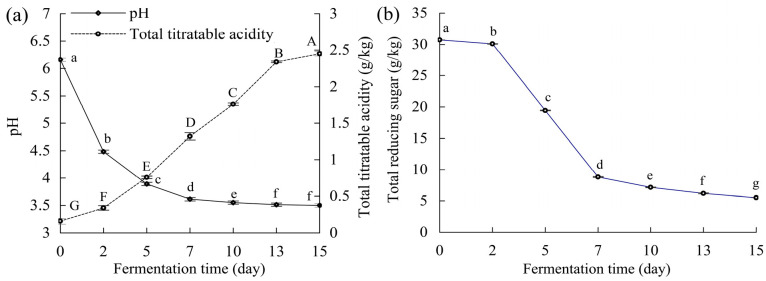
The changes in pH value, total titratable acidity (**a**), and total reducing sugar (**b**) during the 15-day fermentation of Shuidong mustard. Different lowercase (or capital) letters near the index values indicate that the one-way ANOVA of the mean values differs significantly (*p* < 0.05).

Based on the results of the three analysis indices (Figure 1), Shuidong mustard received fermentation under the used conditions because decreased pH value, enhanced level of total titratable acidity (calculated as lactic acid), and reduced content of reducing sugar were observed. In the early fermentation stage (0–7 days), pH values and total titratable acidity showed a rapid decrease (from 6.16 to 3.61) and increase (from 0.16 to 1.32 g/kg) (Figure 1a), respectively, which was closely associated with the rapid growth and metabolic activity of lactic acid bacteria. Meanwhile, total reducing sugar was also reduced quickly (from 30.77 to 8.80 g/kg) at the early fermentation stage (Figure 1b). In the latter fermentation stage (7–15 days), both pH values and total reducing sugar showed a slower extent in data reduction, while total titratable acidity also demonstrated a continuous increase trend. At the end point of mustard fermentation, pH values and total reducing sugar were decreased to 3.15 and 5.50 g/kg, respectively, while total titratable acidity was promoted to 2.45 g/kg. The changing profiles of the three indices were consistent with the growth curve of lactic acid bacteria that is well-known or investigated in the fermented foods. In theory, the changing tendency of reducing sugar and total titratable acidity was ascribed to the proliferation of the natural lactic acid bacteria under 20 g/k table salt usage, which rapidly consumed reducing sugar and accumulated organic acids, leading to a marked decrease in pH and a continuous increase in total titratable acidity. It was thus concluded that under the lower usage of table salt (20 g/kg), lactic acid bacteria could also dominate the fermentation process of Shuidong mustard and generate organic acids through using reducing sugar as the fermentation substrate.

The patterns of acid accumulation, pH decline, and consumption of total reducing sugar had been explored in the microorganism fermentation of Sichuan pickles and northeastern sauerkraut [1,25], while the results from the two studies were consistent with the present one. This result consistency suggested that the core fermentation pathway was not disrupted by the usage reduction in table salt. Both total titratable acidity and pH are the two direct indices to reflect the anaerobic fermentation of vegetables. It was reported that the pH value at the end of sauerkraut fermentation ranged from 3.6 to 4.0, while the total titratable acidity ranged from 4.0 to 5.0 g/L [26]. Additionally, the total titratable acidity of northeastern sauerkraut fermented at a salinity of 3.5% (*w*/*w*) was up to 6 g/kg [25]. Reducing sugar is the primary energy source for microorganisms, and can be metabolized into lactic acid, ethanol, and other metabolites [27]. In northeastern sauerkraut fermented with 25 g/kg table salt, it was observed that the content of total reducing sugar was reduced to 5.2 g/kg [25]. In the fermentation of radish under a higher salt usage (98 g/kg), total reducing sugar had a beginning level of 20.02 g/kg but was decreased gradually during the fermentation [28]. These mentioned studies thereby provided a result support to the present one.

### 3.2. Main Microorganisms Detected in the Fermented Shuidong Mustard

**Figure 2:**
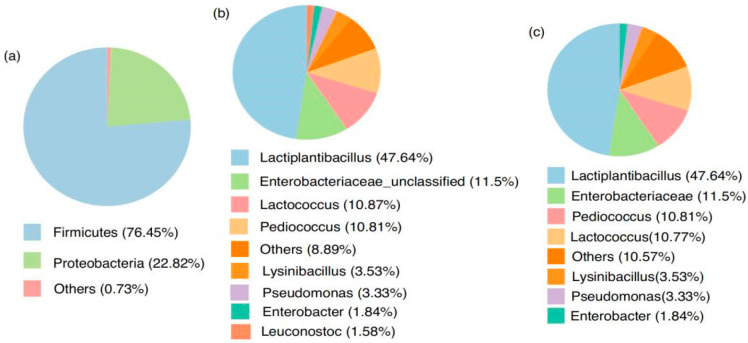
The structures and abundances of the microbial community in the fermented Shuidong mustard (fermentation time of 15 days) characterized at the respective phylum (**a**), genus (**b**), and species (**c**) levels.

The microorganism structure within the fermented Shuidong mustard sample was identified using the 16S rRNA gene sequencing and are now outlined in Figure 2. At the phylum level, the microorganism community was structured by *Firmicutes* (76.45%) and *Proteobacteria* (22.82%) that constituted the predominant taxa, alongside minor fractions (0.73%) (Figure 2a). A further resolution at the genus rank could identify *Lactiplantibacillus* (47.64%), *Enterobacteriaceae* (11.50%), *Lactococcus* (10.87%), and *Pediococcus* (10.81%) as the dominant genera (Figure 2b). To achieve finer taxonomic discrimination, the species-level analysis results also confirmed that *Lactiplantibacillus* (47.64%), *Enterobacteriaceae* (11.50%), *Pediococcus* (10.81%), and *Lactococcus* (10.77%) were the prevailing species (Figure 2c).

When northeastern Chinese cabbage was fermented with a salt content of 20 g/kg, it was found that the predominant phyla included *Proteobacteria* (55.05%), *Bacteroidetes* (40.79%), and *Firmicutes* (1.79%) [29]. Moreover, it was identified that *Firmicutes*, *Proteobacteria*, and *Bacteroidetes* were the dominant genera in Korean kimchi [30], while *Lactobacillus*, *Lactococcus*, and *Pediococcus* were the dominant genera in Yunnan pickles fermented with 24 g/kg table salt [31]. Additionally, *Lactococcus*, *Pediococcus*, and *Flavobacterium* were identified as the dominant genera in Guangxi pickles fermented with 21 g/kg table salt [32]. Notably, *Enterobacter* was detected as the key genus in Shanxi pickles fermented with a salt content of 40 g/kg [33]. It was also reported that *Lactiplantibacillus*, *Pediococcus*, and *Lactococcu*s were dominant species in northeastern sauerkraut fermented with 20 and 30 g/kg table salt usage [34], whereas *Flavobacterium* alongside *Pseudomona*s in fermented Zhejiang mustard were the dominant species [35]. Collectively, these reported results aligned with the outcomes of the present study.

Lactic acid bacteria are the principal and prevailing taxa in vegetable fermentation, owing to their activities in advancing acidification and fermentation. In the present study, both *Lactiplantibacillus* and *Lactococcus* were identified in the fermented Shuidong mustard; however, pathogenic genera were found to exceed the relative abundance of lactic acid bacteria. This might have arisen from the no sterilization before and after the explored mustard fermentation. The investigated mustard samples were exposed to an uncontrolled environment, facilitating the colonization of some undesirable microorganisms. Thus, for industrial-scale production of fermented Shuidong mustard, both sterilization and appropriate packaging after mustard fermentation are necessary to prevent the possible microbial contamination. Further study might focus on these possible chemical and microbial concerns of food safety, including biogenic amines, microbial contamination, and others [36].

### 3.3. The Changes in Several Major and Minor Components in the Fermented Shuidong Mustard

The analysis results (Table 1) showed that the performed fermentation of Shuidong mustard brought about a slight but statistically significant (*p* < 0.05) reduction in water content (from 956.8 to 947.6 g/kg), partly as a result of the table salt usage that introduced the entrance of table salt into vegetable tissues. NaCl and ash contents in the fermented Shuidong mustard were thereof elevated significantly (*p* < 0.05) due to the same reason. Meanwhile, moisture decrease might also be caused partly by water evaporation and tissue exudation, while content increases in NaCl and ash might also be partly induced by moisture loss. For the three minor components, namely vitamin C, total phenols, and total carotenoids, they are nutrients or regarded as nutraceuticals with healthy function in the body; however, they all (especially vitamin C) showed content reduction (*p* < 0.05) after the performed fermentation. More importantly, another two minor components (i.e., nitrite and nitrate) also showed content decreases (*p* < 0.05) after finishing the fermentation. Decreased contents of vitamin C and total phenols might be caused by both chemical oxygen and leaching during the fermentation, while reduced contents of total carotenoids, nitrite, and nitrate might be triggered by the microorganism-mediated biotransformation and leaching [17]. Overall, the fermented Shuidong mustard totally had decreased levels of the five minor components but simultaneously received higher NaCl/ash contents.

NaCl usage in the fermentation of Shuidong mustard ensured the entrance of some Na^+^ and Cl^−^ into vegetable tissues, reasonably causing higher NaCl and ash contents but reduced water content. Vitamin C and phenolic substances as two natural antioxidants are sensitive to oxygen or oxidants, and can undergo oxidative degradation to cause content decrease. It was also reported that vitamin C content in the fermented Indian mustard was decreased after the 7-day fermentation due to its gradual leaching [37]. Other minor components like carotenoids and phenols also have been investigated for their changes in the fermented vegetables. During the fermentation of Polish cabbage and American kale, both total carotenoids and phenols were observed to have content decrease [38,39]. Previous studies also explored the changes in nitrite and nitrate contents during vegetable fermentation. For example, it was found that the detected nitrite content in the pickles fermented with 30 and 60 g/kg table salt was 1.0 mg/kg, while nitrate content of the fermented or pickled kimchi was in a safety level of 1220 mg/kg [40,41]. More importantly, it was demonstrated in a study on American pickles that the performed fermentation caused content decrease in both nitrite and nitrate [40]. Nitrite content less than 20 mg/kg is considered as a safety limit for foods [1]. Thus, the fermented Shuidong mustard had a safety level of nitrite or nitrate, because its nitrite and nitrate contents were less than these contents mentioned in the three studies.

### 3.4. Textural Features of the Fermented Shuidong Mustard

The textural properties of fermented Shuidong mustard were quantified using the TPA technique. The results (Table 2) indicated that the performed fermentation altered the textural profile of Shuidong mustard significantly (*p* < 0.05). Totally, a value reduction was observed across all four indices. In detail, hardness value was decreased from 37.66 to 15.00 g, while chewiness value was declined from 1.35 to 1.23 mJ. Similarly, both springiness and fracturability values were reduced from 0.75 mm and 18.04 g to 0.57 mm and 11.25 g, respectively. The reduced values for the four textural indices were mainly caused by cell wall damage or degradation occurred during the fermentation. The reduction in the four textural indices suggested that the used Shuidong mustard fermentation led to a substantial transformation or, more specially, an improvement in textural characteristics.

Textural assessment in fermented vegetables often utilizes hardness as a critical parameter. The action of these enzymes like pectinase, polygalacturonase, and pectin methylesterase during vegetable fermentation enhances pectin breakdown, thereby resulting in diminished firmness. It is well-known that pectin and pectin-like substances are the necessary components of the cell wall of vegetables, while a degradation of pectin and pectin-like substances surely causes tissue damage and changed textural indices for vegetables [31]. A set of study results about Yunnan pickled peppers, which were fermented under both low (25 g/kg) and high (90 g/kg) table salt conditions, have indicated a reductions in hardness, springiness, and chewiness [31]. Similar results were noted in the explored Hunan mustard [15]. Generally, textural changes are largely driven by lactic acid bacteria, which generates lactic acid and promotes structural disintegration of vegetable tissues, thereby affecting textural features of the fermented vegetables [31]. The fermented Shuidong mustard thus exhibited reduced textural indices and was regarded with improved textural quality. Consumer acceptance of the fermented Shuidong mustard in its texture still needs a further sensory evaluation.

### 3.5. Volatile Substances of the Fermented Shuidong Mustard

A comprehensive profiling of volatile compounds in the fermented Shuidong mustard samples was explored briefly using the HS-SPME/GC-MS technique. In total, 90 volatiles were identified from the fermented Shuidong mustard. Among these detected volatiles, 21 volatiles were classified as the dominant volatile contributors because they constituted 95% (*w*/*w*) of the total volatiles (Table 3). The remaining 69 volatiles, comprising 5% (*w*/*w*) of the total volatiles, were designated as the minor volatile contributors (Table 4). Based on their structural features, the 21 predominant volatiles were chemically categorized into 11 classes, including four alcohols, one acid, one phenolic compound, two heterocyclics, two unsaturated hydrocarbons, four aldehydes, one ketone, two esters, two alkanes, one nitrile derivative, and one peroxide. Meanwhile, these minor volatiles were divided into 12 classes. Quantitative analysis results also revealed that the alcohols constituted the most prevalent class (59.43%) in the dominant volatiles, succeeded by acetic acid (15.26%), esters (5.86%), and aldehydes (5.61%), and then the remaining classes. Notably, both 2-butyl and 3-butenyl isothiocyanates were identified among the major volatile constituents (total content of 5.86%), whereas others, namely isobutyl and ethyl thiocyanates, were detected as the minor components (total content of 0.28%). In the major volatile compounds, ethanol accounted for the highest level, while its production was generally attributed to the alcoholic fermentation mediated by yeasts or certain bacteria under the low-salt condition. Meanwhile, other alcohols like 3-hexenol and cyclobut-1-enylmethanol were also detected, which generally contribute grassy and subtle floral notes [31]. Acetic acid, characterized by a typical sour and pungent note, was the second most abundant volatile component, while its generation was caused by the metabolic activities of acetic acid bacteria or heterofermentative lactic acid bacteria during the fermentation. Both ethanol and acetic acid were also detected in the fermented mustard using the wild-type microorganisms [31]. Aldehydes like pentanal, 3-methylbenzaldehyde, 2,4-heptadienal, and hexaldehyde were generally derived from the oxidative degradation of unsaturated fatty acids during the fermentation. These aldehydes usually have grassy, fatty, and subtle fruity notes, and are regarded as able to enrich the overall flavor profile and complement the typical pungent characteristics imparted by isothiocyanates [15]. Furthermore, among the minor volatile compounds, the greatest diversity was observed in the heterocyclic compounds, by which the roasted and nutty aromas were possibly imparted from the furans and pyrazines [15].

Volatile compounds are vital to the flavor quality of each processed food and thus are efficiently explored by both food scientists and manufactures. Isothiocyanates represent the predominant volatile constituents in the vegetables from cruciferous family, and they also exist in their fermented or processed products. For example, fresh mustard has been documented to harbor two characteristic isothiocyanate substances, namely benzyl and allyl isothiocyanates [42]. It was also reported by Wieczorek and Jeleń that the most abundant volatiles in the 15 raw and cooked *Brassica* cultivars were the sulfur-containing compounds including isothiocyanates [43]. In theory, the thioglucoside compounds in mustard can undergo unavoidable breakdown through three distinct pathways enzymatically, thermally, and chemically, yielding the formation of various isothiocyanate products [8], which contribute to typical aromatic and pungent qualities. Subsequently, various isothiocyanates are identified as the fundamental flavor determinants in the fermented mustard [44]. Sharing same finding conclusion to these mentioned studies, the present study also found that the fermented mustard samples comprised four isothiocyanates, specifically 2-butyl, 3-butenyl, isobutyl, and ethyl isothiocyanates. It was thus suggested that the four isothiocyanates would have their roles in defining the typical flavor profile of processed mustard products. More importantly, isothiocyanates now are considered with several health benefits in the body [45]. The fermented mustard thus might be potential as one of the healthy diets in daily life, because of these identified bioactive components such as isothiocyanates, polyphenols, and carotenoids. However, the flavor quality, especially consumer acceptance of the fermented Shuidong mustard, should be investigated in further studies via a serial of sensory evaluations.

## 4. Conclusions

When the Shuidong mustard was fermented by the wild or native microorganisms at 25 °C for 15 days under the reduced table salt usage (20 g/kg) in water, it received acid production, compositional changes, and, more importantly, textural improvement. Acid production occurred rapidly at the early fermentation stage and then continuously at the latter time *via* utilizing reduced sugar, while the fermented mustard had decreased pH and increased total titratable acidity. Due to table salt usage, the fermented mustard had enhanced NaCl or ash contents. As a result of this fermentation, the fermented mustard had decreased contents in these minor components, like nitrite, nitrate, vitamin C, total phenols, total carotenoids, and received textural improvement by reducing hardness, chewiness, springiness, and fracturability. The fermented mustard also had 90 volatiles (including typical flavor compounds namely 2-butyl, 3-butenyl, isobutyl, and ethyl isothiocyanates), while 21 members comprised 95% of total volatiles. Moreover, the microorganism community of the fermented mustard was structured at respective phylum, genera, or species levels mainly by *Firmicutes* and *Proteobacteria*, *Lactiplantibacillus*, *Enterobacteriaceae*, *Lactococcus*, and *Pediococcus*, or *Lactiplantibacillus*, *Enterobacteriaceae*, *Pediococcus*, and *Lactococcus*. It is thus suggested that this explored mustard fermentation induced significant benefits for Shuidong mustard, including acid production, clear improvement in texture, and reduced nitrite/nitrate contents, and could be used in practical production when being coupled with necessary sterilization and packaging, which might be investigated in further study.

## Figures and Tables

**Table 1:** Major and minor components detected in the Shuidong mustard without or with the fermentation.

Index	0 Days	15 Days
Moisture (g/kg)	956.8 ± 1.4 ^a^	947.6 ± 1.6 ^b^
NaCl (g/kg)	0.54 ± 0.03 ^b^	18.87 ± 0.13 ^a^
Ash (g/kg)	1.04 ± 0.02 ^b^	19.70 ± 0.03 ^a^
Nitrite (mg/kg)	5.72 ± 0.05 ^a^	1.66 ± 0.01 ^b^
Nitrate (mg/kg)	214.06 ± 0.04 ^a^	198.13 ± 0.02 ^b^
Vitamin C (mg/kg)	108.02 ± 0.05 ^a^	12.06 ± 0.04 ^b^
Total phenols (mg/kg)	1058.67 ± 0.36 ^a^	817.62 ± 0.02 ^b^
Total carotenoids (mg/kg)	42.00 ± 0.11 ^a^	10.12 ± 0.06 ^b^

Different lowercase letters as the superscripts after the values in the same row indicate that the one-way ANOVA of the mean values differs significantly (*p* < 0.05).

**Table 2:** Main texture characteristics of the Shuidong mustard without or with the fermentation.

Index	0 Days	15 Days
Hardness (g)	37.66 ± 1.31 ^a^	15.00 ± 0.07 ^b^
Chewiness (mJ)	1.35 ± 0.07 ^a^	1.23 ± 0.04 ^b^
Springiness (mm)	0.75 ± 0.24 ^a^	0.57 ± 0.12 ^b^
Fracturability (g)	18.04 ± 0.11 ^a^	11.25 ± 0.14 ^b^

Different lowercase letters as the superscripts after the values in the same row indicate that the one-way ANOVA of the mean values differs significantly (*p* < 0.05).

**Table 3:** Main volatile compounds detected in the Shuidong mustard fermented for 15 days.

Class	Retention Time (Min)	Compound	Relative Content (%)
Alcohol	5.45	Ethanol	55.99
	15.52	D13-Alcohol	2.64
	10.35	Cyclobut-1-enylmethanol	0.57
	17.13	3-Hexenol	0.23
Acid	19.06	Acetic acid	15.26
Ester	14.00	2-Butyl isothiocyanate	5.19
	19.18	3-Butenyl isothiocyanate	0.67
Aldehyde	6.68	Pentanal	2.16
	23.53	3-Methylbenzaldehyde	1.70
	19.40	2,4-Heptadienal	1.28
	9.00	Hexaldehyde	0.47
Phenol	19.10	Methyl-4-tyramine	2.41
Ketone	6.00	Ethylvinyl ketone	2.25
Alkane	13.00	1,1-Dimethylcyclopropane	1.07
	19.83	Pentadecane	0.69
Heterocyclic compound	6.00	2-Ethylfuran	0.60
	13.01	2-Pentylfuran	0.37
Unsaturated hydrocarbon	17.68	(4z)-1,4-Hexadiene	0.34
	8.50	1,2-Dichloroethane	0.32
Nitrile	14.00	4-Pentenenitrile	0.54
Peroxide	12.21	Heptyl hydroperoxide	0.40

**Table 4:** Minor volatile compounds detected in the Shuidong mustard fermented for 15 days.

Class	Retention Time (Min)	Compound	Relative Content (%)
Heterocyclic compound	19.40	2-(2-Propenyl)-furan	0.19
	26.20	Furan	0.19
	20.40	2-Methoxy-3-(1-methylpropyl)pyrazine	0.16
	20.77	1,5-Dimethyl-1,4-cyclohexadiene	0.16
	33.40	Phytanyl furan	0.04
	29.80	Neophytadiene	0.04
	24.10	N-Benzyloxy-2,2-bis(trifluoromethyl)aziridine	0.03
	4.46	2-Methylfuran	0.01
	26.76	Formamide	0.01
	3.26	2,4-Hexadiene, (2e,4z)-	0.01
Aldehyde	17.45	4-Isopropylidene-2-methyl-cyclopentan-1-al	0.22
	9.00	Propionaldehyde	0.19
	14.40	Acrolein	0.19
	8.10	Crotonaldehyde	0.05
	25.51	4-Ethylbenzaldehyde	0.04
	14.40	Octylaldehyde	0.02
	20.50	Trans-2-decenal	0.02
	4.53	Butyraldehyde	0.01
Alkane	17.25	n-Tetradecane	0.17
	8.56	n-Undecane	0.16
	14.00	3,7-Dimethylundecane	0.05
	23.15	1-Iodo-2-methylundecane	0.05
	29.73	4-Trifluoroacetoxytridecane	0.02
	25.86	1-Chloro-5-methyl-hexane	0.01
	12.68	2,7,10-Trimethyl-dodecane	0.01
	30.75	Nonyl cyclopropane	0.01
	7.01	2,6-Dimethylnonane	0.01
	10.36	4,7-Dimethylundecane	0.01
	10.70	2,3,6-Trimethyl decane	0.01
	32.11	1,2-Epoxyhexadecane	0.01
	11.43	2,4-Dimethylundecane	0.01
	11.51	4,4-Dimethyl-undecane	0.01
	14.90	4,6-Dimethylundecane	0.01
Phenol	37.35	2,4-Di-tert-butylphenol	0.20
	34.98	3-Ethylphenol	0.13
	34.00	8-Dodecenol	0.01
Alcohol	15.15	Cyclopropaneethanol	0.11
	18.55	1-Heptanol	0.10
	8.00	n-Propanol	0.06
	9.81	Allyl alcohol	0.02
	30.53	Hexahydrofarnesol	0.01
Ester	15.50	Isobutyl isothiocyanate	0.19
	13.70	Ethyl caproate	0.17
	5.14	Butan-2-yl cyanate	0.17
	14.25	Methyl thiocyanate	0.16
	36.05	Phenylethyl isothiocyanate	0.11
	3.55	Methyl acetate	0.09
	10.40	Ethyl valerate	0.04
	28.23	Dodecanoic acid, ethyl ester	0.03
	42.53	Methyl linolenate	0.03
	32.03	Methyl diethyldithiocarbamate	0.03
	11.45	n-Amyl acetate	0.01
	11.25	Ethyl crotonate	0.01
Polycyclic aromatic hydrocarbon	10.58	4-Xylene	0.27
	16.00	1,2,3-Trimethylbenzene	0.03
	18.45	1,2,4,5-Tetramethylbenzene	0.02
	10.25	Ethylbenzene	0.02
	8.13	Toluene	0.01
	6.38	5,5-Dimethyl-1-ethyl-1,3-Cyclopentadiene	0.01
	27.25	2,5-Dimethyl-2-undecene	0.01
	17.03	Cyclopropylbenzene	0.01
Unsaturated hydrocarbon	13.50	Styrene	0.22
	29.80	Neophytadiene	0.08
Nitrile	7.36	Acetonitrile	0.27
	14.36	3-Methyl-2-butenenitrile	0.02
Peroxide	15.68	8-Oxabicyclo(5.1.0)octane	0.20
Sulfide	8.85	Dimethyl disulfide	0.15
Ketone	33.95	Phytone	0.06
	27.51	2-Tridecanone	0.04

## Data Availability

The original contributions presented in this study are included in the article. Further inquiries can be directed to the corresponding authors.
